# The complete chloroplast genome sequence of *Sinolimprichtia alpina* var. *dissecta* (Apiaceae)

**DOI:** 10.1080/23802359.2020.1862001

**Published:** 2021-03-30

**Authors:** Chuan-Jiang Liao, Liu Qun, Yu-Sheng Xu, Zhi-Lin Jiang, Cheng-Gui Zhang

**Affiliations:** a Institute of Entomoceutics Research, National-Local Joint Engineering Research Center of Entomoceutics, Dali University, Dali, China; b School of Life Sciences, Yunnan Normal University, Kunming, China; c Hunan Agricultural University, Changsha, China; d Institute of Agricultural and Garden Technology, Puer University, Puer, China; e Institute of Comparative Study of Traditional Materia Medica, Institutes of Integrative Medicine, Fudan University, Shanghai, China

**Keywords:** *Sinolimprichtia alpina* var. *dissecta*, alpine plants endemism chloroplast genome sequence

## Abstract

*Sinolimprichtia alpina* var. *dissecta* is a plant variety which is characterized from *S. alpina* var. *alpina* in possessing characteristic, highly dissected bracteoles. In the current study, we have sequenced the complete chloroplast genome of *S. alpina* var. *dissecta* using the Illumina sequencing platform. The chloroplast genome is 156,719 bp in length, consisting of a LSC region of 95,625 bp, a SSC region of 10,500 bp, and a pair of inverted repeats (IR) regions of 25,297 bp. The GC content was 37.7%. A total of 126 unique genes were identified, including 81 protein-coding genes, 37 tRNA genes and 8 rRNA genes. Phylogenetic analysis based on 28 chloroplast genomes indicates that *S. alpina* var. *dissecta* is most closely related to *Pterygopleurum neurophyllum*.

*Sinolimprichtia alpina* var. *dissecta* is a plant variety which is characterized from *S. alpina* var. *alpina* in possessing characteristic, highly dissected bracteoles. This species is distributed in Qinghai, SW Sichuan, SE Xizang, NW Yunnan occurring at altitudes from 3300 to 5000 meters above sea level. We report the first complete chloroplast genome of *S. alpina* var. *dissecta* using Illumina sequencing and resolve the phylogenetic position and characterize the genomic structure.

The plant sample of *S. alpina* var. *dissecta* used for sequencing was collected from Ganzi County, Ganzi Tibetan Autonomous Prefecture, Sichuan Province, China (31°49′16.94″N, 100°15′45.41″E). The voucher specimens are archived at the Herbarium of Kunming Institute of Botany, Chinese Academy of Sciences (KUN, KUN1490887). Total genomic DNA was extracted to construct a library for sequencing by using Illumina sequencing methods at the Beijing Novogene Bioinformatics Technology Center. The clean data of *S. alpina* var. *dissecta* was *de novo* assembled using NOVOPlasty v3.1 (Dierckxsens et al. 2017) and GetOrganelle pipeline (Jin et al. 2018). Then, the resulting contigs were identified and rearranged by mapping to reference genome *Pleurospermum camtschaticum*. Assembled plastid genome annotation was conducted using Geseq in Chlorobox web service (Tillich et al. 2017), with manual corrections for start and stop codons using Geneious v.9.0.2 (Kearse et al. 2012). The annotated plastid genome sequence was submitted to Genbank (MW221263).

The complete chloroplast genome of *S. alpina* var. *dissecta* is 156,719 bp in length, with a typical quadripartite structure, and contains a large single-copy (LSC) region of 95,625 bp, a small single-copy (SSC) region of 10,500 bp, a pair of inverted repeats (IR) regions of 25,297 bp. The overall GC content was 37.7%. A total of 126 unique genes, containing 81 protein-coding genes, 37 tRNA genes and eight rRNA genes.

We conducted a phylogenetic analysis to investigate the phylogenetic relationships among the whole chloroplast genome sequence of *S. alpina* var. *dissecta* and that of other 27 species of Apioideae in the NCBI GenBank database. We used maximum likelihood (ML) in RAxML8.0 with 1000 bootstrap replicates to construct a phylogenetic tree (Stamatakis 2014). In the ML tree, our results showed that *S. alpina* var. *dissecta* was most closely related to *P. neurophyllum* with strong support (Figure 1). The chloroplast genome of *S. alpina* var. *dissecta* will provide useful genomic information for future studies on genetic diversity in this group of rarely studied plants.

**Figure 1.**
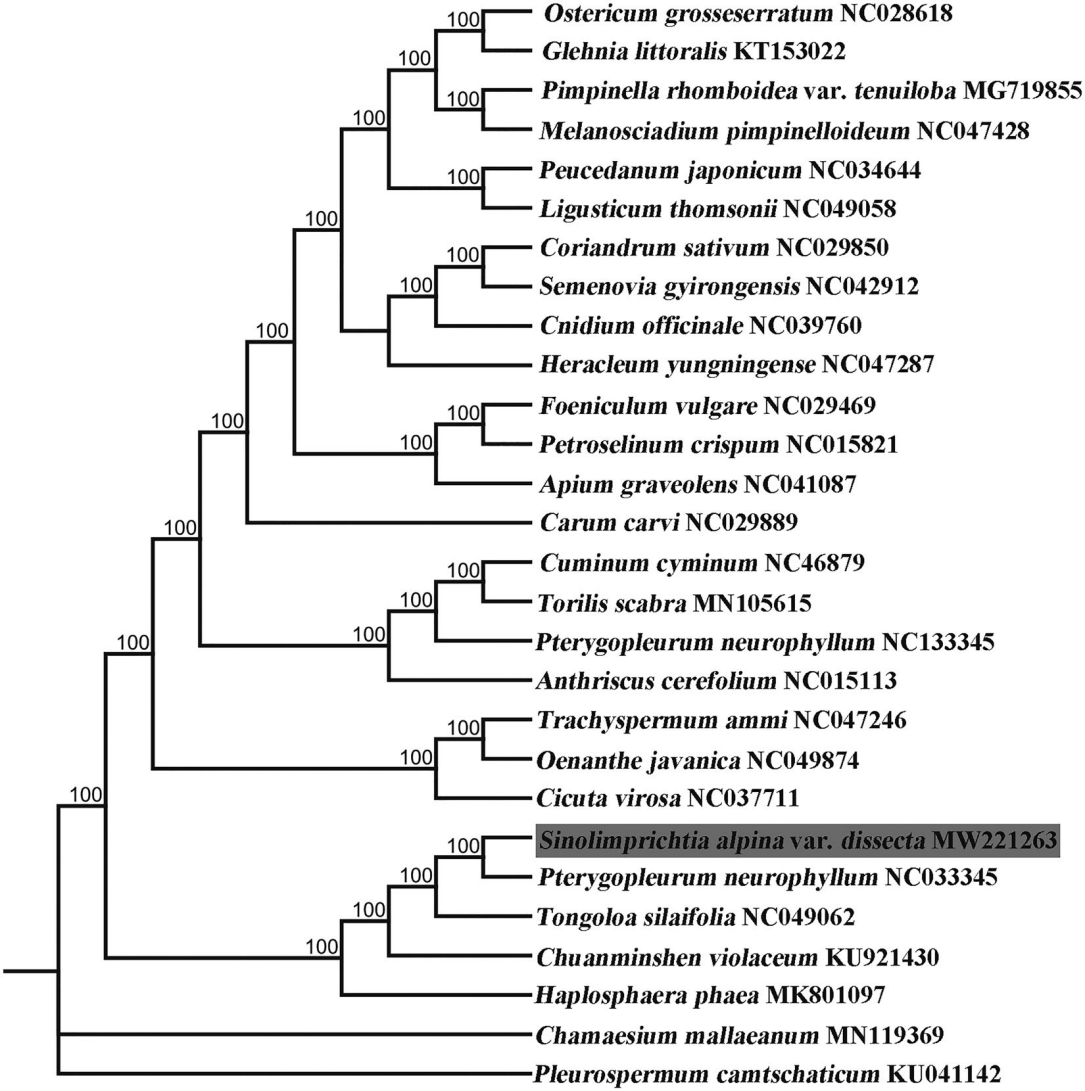
Maximum-likelihood phylogenetic tree based on the chloroplast genome sequences. Numbers at nodes represent bootstrap support values.

## Data Availability

The data that support the findings of this study are available in [GeneBank] at [heeps:www.ncbi.nlm.nih.gov/genbank/], reference number [MW033345], and the SRA data can be cited for in [GeneBank] at [https://www.ncbi.nlm.nih.gov/sra/PRJNA675925], reference number [PRJNA675925].
